# Parallel process evaluation using a proposed framework for the design and reporting of process evaluations for cluster-randomised trials of complex interventions

**DOI:** 10.1186/1745-6215-14-S1-O88

**Published:** 2013-11-29

**Authors:** Aileen Grant, Tobias Dreischulte, Bruce Guthrie

**Affiliations:** 1 University of Dundee, Dundee, UK; 2 NHS Tayside, Dundee, UK

## 

Process evaluations are recommended to open the ‘black box’ of complex interventions evaluated in trials, but there is limited guidance to help with design, with most guidance focused on the use of qualitative methods rather than processes to evaluate. We have developed a framework that identifies a range of candidate processes that an evaluation *could* examine to understand cluster-randomised intervention implementation and maintenance. These include processes involving clusters (recruitment, delivery of the intervention to the cluster, adoption by the cluster), involving the individuals on whom outcomes are measured (reach, delivery of the intervention to the individual, response of the individual), maintenance, and unintended consequences. Researchers will often have to choose what to focus on because of resource constraints, the framework aims to help researchers make explicit their choices of research questions and methods.

We will illustrate application of the framework to the design and conduct of a parallel process evaluation of the Data-driven Quality Improvement in Primary Care trial, which evaluates a complex intervention to improve prescribing safety in 40 general practices. This mixed method process evaluation includes a dominant case study approach in a purposeful sample of practices and hypothesis–testing quantitative analysis of data from all practices examining how key processes are associated with variation in outcome between practices. We will present analysis of qualitative data from the 10 case study practices participating (6 reducing and 4 not reducing targeted prescribing), and focus groups with patients who have had their medication changed as a result of the intervention.

